# Time- and posture-specific management of severe supine hypertension and neurogenic orthostatic hypotension in diabetic autonomic neuropathy: A case report

**DOI:** 10.1097/MD.0000000000051516

**Published:** 2026-11-06

**Authors:** Petra Šinigoj Porčnik, Amela Kabaklić

**Affiliations:** a Division of Internal Medicine, Department of Hypertension, University Medical Centre Ljubljana, Dr. Peter Držaj Hospital, Ljubljana, Slovenia; b Department of Internal Medicine, Faculty of Medicine, University of Ljubljana, Ljubljana, Slovenia.

**Keywords:** blood pressure monitoring, case report, diabetic autonomic neuropathy, neurogenic orthostatic hypotension, supine hypertension

## Abstract

**Rationale:**

Coexistence of neurogenic orthostatic hypotension with supine or nocturnal hypertension creates a therapeutic challenge because treatment of either component may aggravate the other. This case illustrates a clinical management framework based on characterization of the full hemodynamic phenotype, repeated reassessment, and treatment adaptation according to posture and time of day, integrating postural blood pressure (BP) responses, 24-hour BP monitoring, symptoms, functional status, comorbidities, and safety findings.

**Patient concerns:**

A 63-year-old man with hypertension and insulin-treated type 2 diabetes presented with orthostatic dizziness and presyncope. Antihypertensive therapy was intensified for elevated seated BP. After intensification, upright tolerance worsened, with recurrent syncope, a rib fracture, and loss of functional independence. Symptoms remained disabling despite antihypertensive withdrawal and nonpharmacological measures.

**Diagnoses:**

Supine-to-seated-to-standing assessment showed a BP fall from 197/99 mm Hg supine to 122/81 mm Hg seated and 79/56 mm Hg standing, with presyncope and heart rate increasing from 72 to 86 beats/min. Supine measurements and 24-hour BP monitoring documented severe supine and nocturnal hypertension, reverse dipping, and mean asleep BP 203/107 mm Hg. Autonomic testing demonstrated combined cardiovagal and adrenergic dysfunction, supporting neurogenic orthostatic hypotension due to diabetic autonomic neuropathy.

**Interventions:**

Management was tailored to upright and recumbent periods. A removable transdermal nitroglycerin patch was used overnight. Nonpharmacological measures included repeated encouragement to increase fluid intake, but daily intake generally remained below 2 L. Clinical assessment suggested relative volume depletion. Low-dose morning fludrocortisone was introduced cautiously despite severe supine/nocturnal hypertension. After upright tolerance improved, morning lercanidipine was added. Subsequent 24-hour BP monitoring showed a more balanced profile.

**Outcomes:**

Orthostatic symptoms and upright tolerance improved. No further syncope or falls occurred, and functional independence improved. Later 24-hour BP monitoring showed reduced nocturnal predominance, with mean awake/asleep BP 164/86 and 165/89 mm Hg, respectively. Mild peripheral edema was managed with intermittent torasemide.

**Lessons:**

This case illustrates how integrating postural BP responses with 24-hour BP patterns can identify competing hemodynamic phenotypes that may require different management during upright and recumbent periods. Repeated reassessment and individualized treatment adaptation according to the documented BP pattern and clinical context may help balance competing risks of upright hypoperfusion and supine/nocturnal hypertension.

## 1. Introduction

Diabetic autonomic neuropathy may involve the cardiovascular autonomic system, leading to impaired cardiovagal and sympathetic vasomotor regulation and compromising the compensatory heart rate (HP) and vasoconstrictor responses required to maintain blood pressure (BP) during postural change.^[1,2]^ In advanced autonomic dysfunction, neurogenic orthostatic hypotension (nOH) may coexist with neurogenic supine hypertension (SH) and abnormal nocturnal BP profiles, producing marked BP variation over the 24-hour period.^[3,4]^ This phenotype is clinically important because orthostatic hypotension may cause cerebral hypoperfusion, presyncope and syncope, with consequent falls and injuries.^[5,6]^ Orthostatic hypotension may also compromise perfusion of other organs and tissues, resulting in symptoms such as dyspnea or chest discomfort in some patients.^[5,6]^ Sustained supine or nocturnal hypertension may contribute to end-organ damage and cardiovascular and cerebrovascular risks, although its independent prognostic contribution remains uncertain.^[4]^

Management of coexisting nOH and SH is particularly challenging because interventions intended to improve upright BP may aggravate SH, whereas treatment directed at SH may worsen orthostatic symptoms or compromise upright perfusion.^[3,7]^ Reliance on seated BP may obscure clinically relevant postural BP changes and contribute to treatment intensification without recognition of coexisting orthostatic hypotension.^[3,7,8]^ Postural BP assessment and home or 24-hour BP monitoring may help define the BP pattern before antihypertensive treatment is intensified.^[3,7,8]^ Therapeutic decisions should be individualized, integrating symptom burden, functional impact, postural BP responses, out-of-office BP patterns, relevant comorbidities, and treatment-related adverse effects.^[3,8]^ Autonomic testing should be considered when a neurogenic cause is suspected or requires confirmation.^[3]^

This report describes a patient with diabetic autonomic neuropathy, severe neurogenic orthostatic hypotension, and concomitant supine and nocturnal hypertension whose treatment was individualized according to posture and time of day. The value of this case lies in illustrating a systematic, physiology-informed approach to clinical decision-making in the presence of competing hemodynamic risks. Because available evidence does not define a single optimal treatment regimen for nOH with coexisting SH, treatment adjustment was guided by symptoms, changes in functional status, postural hemodynamics, serial 24-hour BP monitoring, relevant comorbidities, and safety surveillance.^[3,6,7]^

## 2. Case presentation

### 2.1. Patient information and clinical findings

A 63-year-old former smoker presented with progressive orthostatic dizziness, presyncope, recurrent syncope, fatigue, and a marked decline in independent daily functioning. His relevant medical history included long-standing arterial hypertension, hypertensive heart disease, hyperlipidemia, and insulin-treated type 2 diabetes mellitus complicated by diabetic retinopathy. Family history was noncontributory.

Before referral to the specialist hypertension unit, management had been guided primarily by persistently elevated seated BP. At that stage, coexisting orthostatic hypotension had not yet been recognized. Antihypertensive therapy was therefore intensified and consisted of perindopril 4 mg once daily, indapamide 1.25 mg once daily, lacidipine 2 mg once daily, and nebivolol 5 mg once daily. Concomitant treatment included acetylsalicylic acid, atorvastatin, pantoprazole, and premixed insulin aspart administered twice daily.

After intensification of antihypertensive treatment, the patient experienced substantial worsening of orthostatic intolerance. The patient experienced recurrent syncope, including an episode resulting in a traumatic rib fracture. This sequence highlighted the central clinical conflict of antihypertensive management based primarily on seated BP without consideration of the full BP profile across posture and time of day, as treatment intensification for elevated seated BP was followed by worsening upright tolerance. Fear of further falls progressively restricted his activity, and he required assistance from his wife with daily activities, including personal hygiene and applying compression stockings, as well as assistance with standing up and walking. He reported substantial psychological distress and loss of confidence.

The clinical deterioration prompted withdrawal of the antihypertensive regimen and initiation of nonpharmacological measures, including increased oral fluid and salt intake, compression therapy, head-of-bed elevation, and education regarding gradual postural transitions. Despite these interventions, severe orthostatic symptoms and functional impairment persisted, and the patient was admitted to a specialist hypertension unit for further evaluation and treatment.

At admission, supine BP was markedly elevated after 5 minutes of supine rest and remained without substantial change on repeat measurement after 10 minutes, when it was 233/102 mm Hg. Body weight was 84.0 kg, body mass index was 30.1 kg/m^2^, and waist circumference was 103 cm. Clinical examination revealed a systolic murmur over the aortic valve but no evidence of overt heart failure or peripheral edema.

Because the clinical history indicated a high likelihood of severe orthostatic hypotension, bedside postural BP assessment was performed using a cautious supine-to-seated-to-standing sequence rather than immediate transition from the supine to the standing position. BP decreased from 197/99 mm Hg with a heart rate of 72 beats/min while supine to 122/81 mm Hg with a heart rate of 71 beats/min while seated, and subsequently to 79/56 mm Hg with a heart rate of 86 beats/min on standing. Standing was poorly tolerated because of presyncopal symptoms. The magnitude of the BP reduction, together with the limited compensatory increase in heart rate, raised suspicion of neurogenic orthostatic hypotension.

### 2.2. Diagnostic assessment

Initial laboratory testing showed no anemia, iron deficiency, vitamin B12 deficiency, or folate deficiency. Serum creatinine was 87 µmol/L, with an estimated glomerular filtration rate of 81 mL/min/1.73 m^2^. Serum electrolytes and thyroid function were within the reference ranges. N-terminal pro–B-type natriuretic peptide was also within the reference range, and there was no clinical evidence of overt heart failure. Glycated hemoglobin was 6.5%, and lipid values were within the recommended targets during statin treatment.

Secondary and potentially reversible contributors to severe BP instability were systematically assessed. A low-dose adrenocorticotropic hormone stimulation test showed an appropriate cortisol response. Hemodynamically significant renal artery stenosis was excluded, and adrenal endocrine screening did not provide biochemical evidence of primary aldosteronism or another clinically relevant adrenal cause of hypertension. Urinary catecholamine measurements were within the reference range on 2 occasions.

Baseline 24-hour BP monitoring, performed after withdrawal of antihypertensive therapy and implementation of nonpharmacological measures, demonstrated marked nocturnal hypertension and a reverse-dipping pattern (Fig. 1A). Mean 24-hour BP was 188/99 mm Hg, mean awake BP was 180/94 mm Hg, and mean asleep BP was 203/107 mm Hg, corresponding to a nocturnal BP increase of 13% systolic and 14% diastolic.

**Figure 1.**
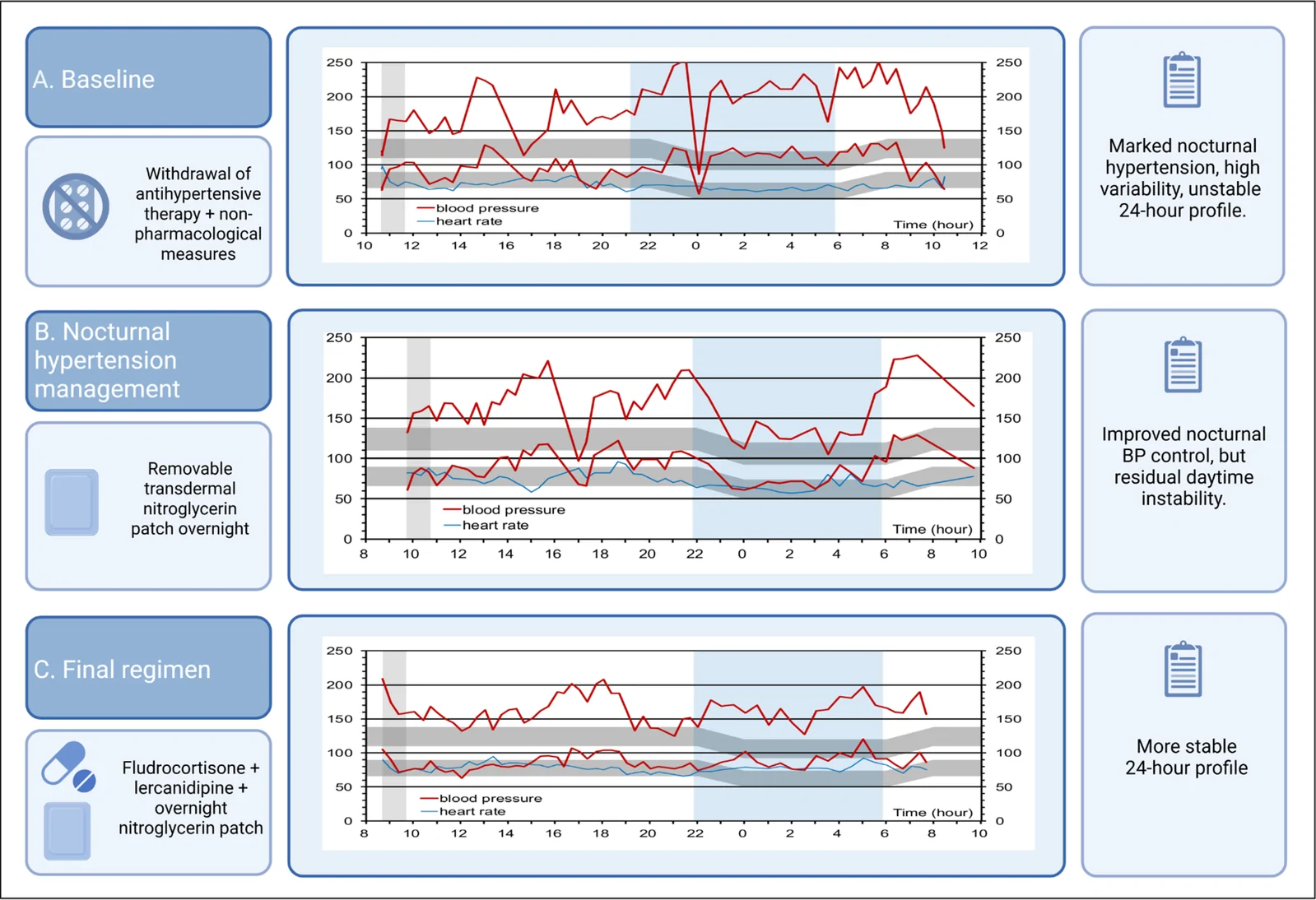
Serial 24-hour blood pressure monitoring during treatment adjustment. Panel (A) shows marked nocturnal hypertension with a reverse-dipping pattern after withdrawal of antihypertensive therapy and implementation of nonpharmacological measures (24-hour BP 188/99 mm Hg; awake BP 180/94 mm Hg; asleep BP 203/107 mm Hg; nighttime BP rise + 13/+14%). Panel (B) shows improved nocturnal BP control after the introduction of an overnight removable transdermal nitroglycerin patch, although daytime BP remained elevated and variable (24-hour BP 160/89 mm Hg; awake BP 172/95 mm Hg; asleep BP 137/75 mm Hg; nighttime BP fall − 20/−21%). Panel (C) shows a more stable 24-hour BP profile after morning fludrocortisone, morning lercanidipine, and continued overnight nitroglycerin, with reduced daytime BP variability and a smaller day-night BP difference (24-hour BP 164/87 mm Hg; awake BP 164/86 mm Hg; asleep BP 165/89 mm Hg; nighttime BP rise + 1/+3%). BP = blood pressure. Figure layout created in BioRender. Kabaklić, A. (2026). **Alt text:** Serial 24-hour blood pressure profiles showing marked nocturnal hypertension with reverse dipping at baseline, improved nocturnal control after overnight nitroglycerin, and a more balanced 24-hour profile after combined treatment with morning fludrocortisone, morning lercanidipine, and overnight nitroglycerin.

Formal autonomic nervous system testing, performed and interpreted by a neurologist, demonstrated markedly reduced heart rate variability during deep breathing and the Valsalva maneuver, consistent with cardiovagal dysfunction. BP recovery during the Valsalva maneuver was inadequate and delayed, supporting adrenergic dysfunction. Head-up tilt testing demonstrated an orthostatic systolic BP reduction of 40 mm Hg, with coexisting supine BP of 154/100 to 110 mm Hg. These findings supported combined cardiovagal and adrenergic impairment and were considered compatible with nOH caused by diabetic autonomic neuropathy (Fig. 2).

**Figure 2.**
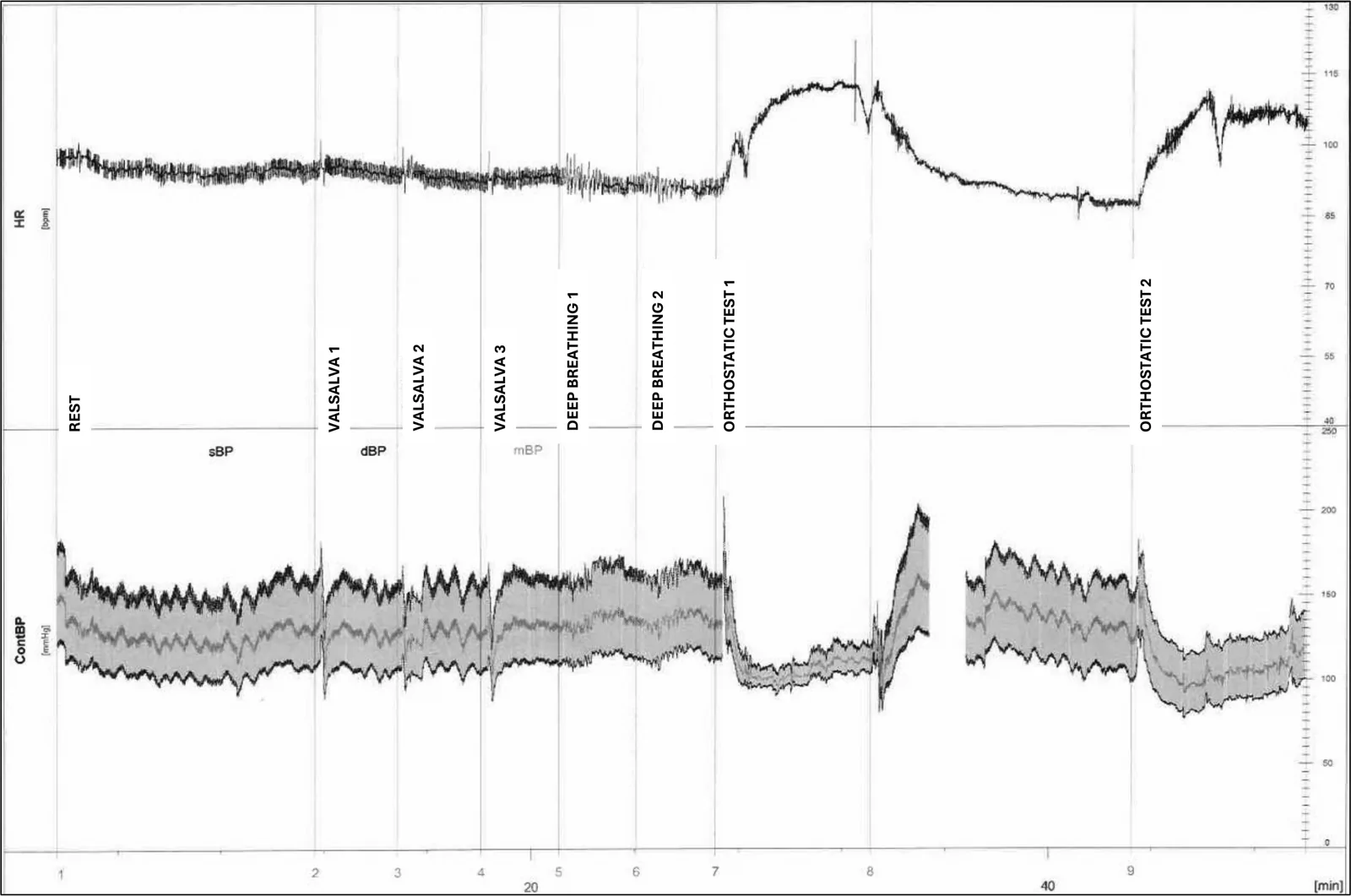
Formal autonomic nervous system testing. Continuous HR and BP recording during autonomic testing. HR variability during deep breathing and the Valsalva maneuver was markedly reduced, supporting cardiovagal dysfunction. BP recovery during the Valsalva maneuver was inadequate and delayed, consistent with adrenergic dysfunction. Orthostatic testing demonstrated OH, with a systolic BP fall of 40 mm Hg, together with coexisting supine hypertension, with supine BP 154/100 to 110 mm Hg during testing. A brief signal interruption reflects technical signal loss during recording. Overall, the findings supported diabetic autonomic neuropathy as the most likely cause of neurogenic OH with coexisting supine hypertension. BP = blood pressure, ContBP = continuous blood pressure, HR = heart rate, OH = orthostatic hypotension, SH = supine hypertension. **Alt text:** Continuous autonomic testing tracings show reduced HR variability during deep breathing and the Valsalva maneuver, impaired BP recovery during the Valsalva maneuver, orthostatic hypotension during orthostatic testing, and coexisting supine hypertension.

The patient reported fatigue after meals; however, a postprandial BP decrease was not objectively documented.

Transthoracic echocardiography demonstrated hypertensive heart disease with preserved left ventricular systolic function (left ventricular ejection fraction, 56%), mild diastolic dysfunction, and mild aortic stenosis (maximum aortic valve velocity, 2.4 m/s; mean transvalvular gradient, 15 mm Hg). Carotid ultrasonography demonstrated nonobstructive atherosclerosis. Twenty-four-hour electrocardiographic monitoring showed sinus rhythm, a mean heart rate of 83 beats/min, first-degree atrioventricular block, and a brief supraventricular tachycardia episode lasting approximately 2 seconds, without evidence of a clinically relevant arrhythmia to account for syncope.

Computed tomography of the head demonstrated old lacunar ischemic lesions. No clinical features suggested a primary neurodegenerative autonomic disorder. The cerebrovascular findings were considered clinically relevant when selecting treatments that could increase supine BP.

The patient also underwent assessment for obstructive sleep apnea. The apnea–hypopnea index was 26 events/h and increased to 39 events/h in the supine position. Mean oxygen saturation was 94%, with nadir oxygen saturation of 79%, consistent with moderate obstructive sleep apnea. Initiation of continuous positive airway pressure therapy was deferred to outpatient follow-up because immediate inpatient initiation was not available.

The overall findings established a diagnosis of severe nOH due to diabetic autonomic neuropathy, with coexisting severe supine and nocturnal hypertension.

The overall diagnostic and therapeutic framework of this case is summarized in Figure 3.

**Figure 3.**
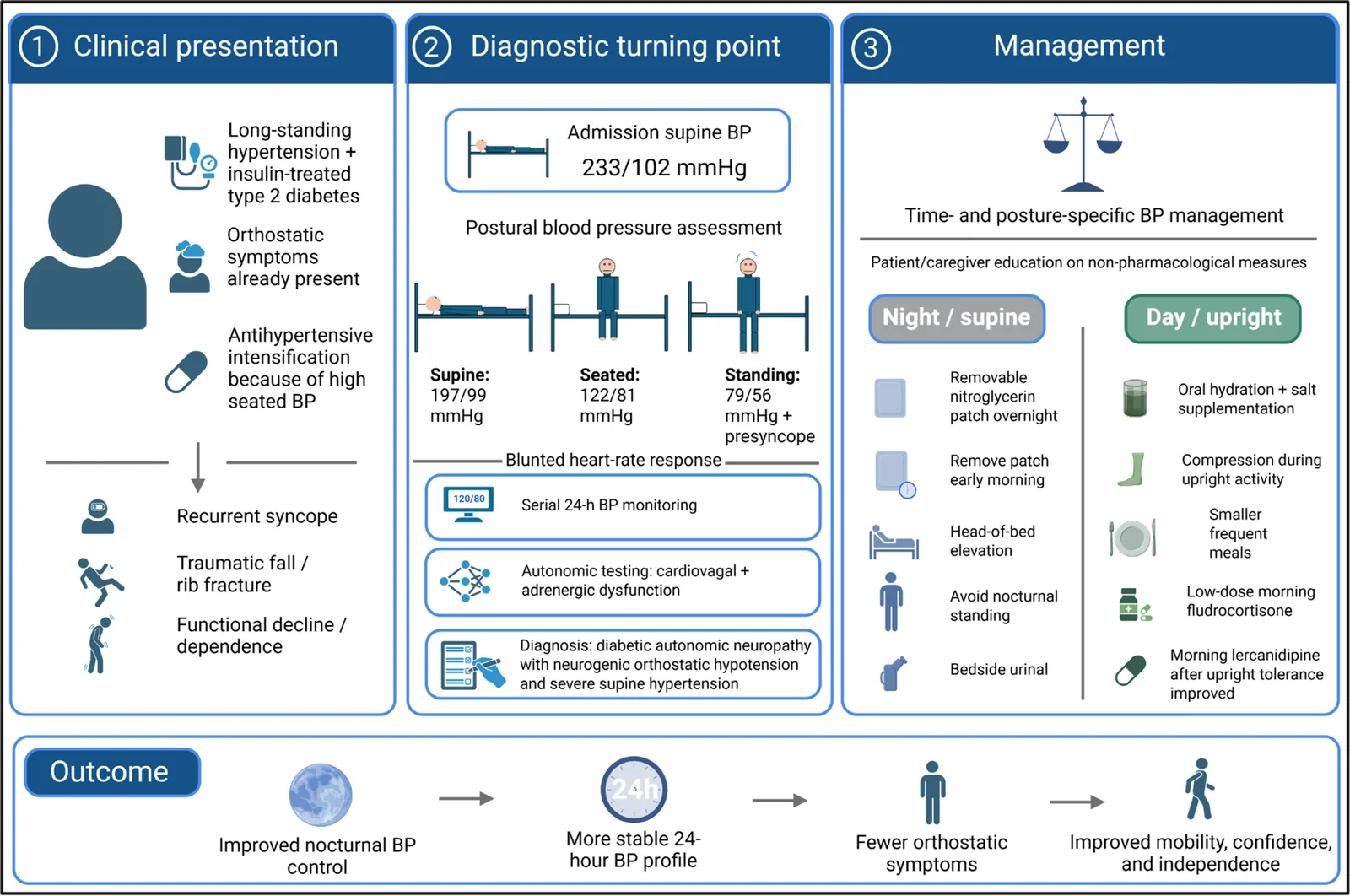
Time- and posture-specific management of bidirectional blood pressure dysregulation in diabetic autonomic neuropathy. The figure summarizes the diagnostic and therapeutic concept used in this case. Severe supine/nocturnal hypertension and upright hypoperfusion were first recognized through postural BP assessment and autonomic testing. Management was then individualized according to time of day and posture, with nocturnal BP lowering, head-of-bed elevation, avoidance of nocturnal standing, support of upright tolerance, and serial 24-hour BP monitoring to assess treatment response and guide treatment adjustment. BP = blood pressure. Created in BioRender. Kabaklić, A. (2026). **Alt text:** Conceptual overview of time- and posture-specific management of coexisting supine/nocturnal hypertension and upright hypoperfusion.

### 2.3. Therapeutic intervention

Treatment was individualized according to posture, time of day, symptom burden, functional status, the 24-hour BP profile, relevant comorbidities, and anticipated treatment-related risks. The therapeutic objective was not uniform BP normalization, but rather improvement of upright perfusion and functional status while limiting exposure to extreme supine and nocturnal hypertension.

The previous antihypertensive regimen had been withdrawn before admission. The patient and his wife received structured education regarding oral hydration, salt supplementation, smaller and more frequent meals, compression garments during upright activity, head-of-bed elevation, gradual postural transitions, physical counter-maneuvers, and avoidance of prolonged standing.

Pharmacological treatment was introduced sequentially and adjusted according to the patient’s clinical response and evolving hemodynamic profile.

Captopril 12.5 mg was initially introduced in the evening but aggravated orthostatic symptoms. Diltiazem 30 mg was then introduced separately and was likewise not tolerated because of worsening orthostatic symptoms. A removable transdermal nitroglycerin patch, 10 mg/24 h, was therefore applied overnight from 22:00 to 06:00 and removed before the first morning mobilization. To reduce the risk of nocturnal hypotension and falls while the patch was active, the patient was instructed to avoid standing during the night and to use a bedside urinal.

Following introduction of the overnight nitroglycerin patch, repeat monitoring showed a mean 24-hour BP of 160/89 mm Hg, a mean awake BP of 172/95 mm Hg, and a mean asleep BP of 137/75 mm Hg (Fig. 1B). The nocturnal pattern changed from reverse dipping to a systolic and diastolic decrease of 20% and 21%, respectively. Although nocturnal BP control improved, daytime BP remained elevated, and postural BP measurements continued to show clinically relevant orthostatic variability.

Despite repeated encouragement to increase fluid intake, daily oral fluid intake generally remained below 2 L. Bedside volume assessment also included qualitative assessment of the inferior vena cava. These findings raised clinical concern for relative volume depletion. In the absence of overt heart failure and with N-terminal pro–B-type natriuretic peptide within the reference range, a cautious low-dose therapeutic trial of fludrocortisone, 0.1 mg once daily in the morning, was initiated. Monitoring focused on orthostatic symptoms, upright tolerance, weight change, signs of fluid retention, serum electrolytes, supine BP, and clinical evidence of heart failure. The need for ongoing fludrocortisone treatment was reassessed during follow-up according to clinical response and safety findings.

Midodrine was deferred because severe supine and nocturnal hypertension, together with previous lacunar ischemic cerebrovascular lesions, raised concern that additional α1-adrenergic vasoconstriction could further increase recumbent BP. Droxidopa was not available for clinical use in our setting.

As upright tolerance improved, the therapeutic focus expanded to reducing the remaining daytime hypertensive burden. Lercanidipine 10 mg once daily in the morning was introduced cautiously during outpatient follow-up. Captopril was prescribed as the first rescue antihypertensive treatment for marked BP elevation, initially at 12.5 mg when standing BP exceeded 180/100 mm Hg, with an additional 12.5 mg administered as needed after BP reassessment. The patient was instructed to reassess BP after captopril administration and to use sublingual nitroglycerin spray only if marked BP elevation persisted. Sublingual nitroglycerin spray was not prescribed for anginal symptoms. Given the patient’s profound orthostatic hypotension, rescue treatment was to be administered while lying down, with avoidance of standing after administration because of the risk of an excessive BP fall, syncope, falls, and injury.

### 2.4. Follow-up and outcomes

Clinical improvement became evident during the 5-week hospitalization. Orthostatic symptoms decreased, standing and walking were better tolerated, and the patient gradually regained confidence in mobilization and basic activities. Body weight increased from 84.0 kg at admission to 85.4 kg at discharge.

At the first outpatient follow-up, the patient reported improved well-being, no recurrent syncope or falls, and greater independence in daily activities. He reported close adherence to the prescribed nonpharmacological measures. During safety monitoring of volume-expanding therapy, body weight had increased to 86.0 kg, and mild peripheral edema subsequently developed. These were considered clinically relevant safety findings. Torasemide 5 mg was prescribed as needed and was taken only intermittently. Serum electrolytes remained within the reference ranges. Fludrocortisone 0.1 mg once daily and the overnight transdermal nitroglycerin patch were continued.

Following the addition of morning lercanidipine and continuation of morning fludrocortisone and overnight nitroglycerin, monitoring demonstrated attenuation of the previous extreme nocturnal predominance and a smaller day–night difference, although BP remained elevated (Fig. 1C). Mean 24-hour BP was 164/87 mm Hg, mean awake BP was 164/86 mm Hg, and mean asleep BP was 165/89 mm Hg.

At the last documented outpatient assessment, approximately 4.5 months after hospital discharge, body weight remained stable at 86.0 kg. Continuous positive airway pressure therapy had been initiated during follow-up. No further syncope or falls were reported during this period.

Overall, treatment response was assessed across complementary clinical and hemodynamic domains: orthostatic symptoms, upright tolerance, recurrence of syncope or falls, functional independence, postural BP measurements, serial 24-hour BP profiles, and surveillance for adverse effects of volume-expanding and BP-lowering therapy. This integrated follow-up was used to guide ongoing treatment adjustment rather than relying on a single BP target.

## 3. Discussion

This case illustrates the diagnostic and therapeutic complexity of severe nOH coexisting with supine and nocturnal hypertension in diabetic autonomic neuropathy. Impaired cardiovagal and sympathetic vasomotor regulation may result in inadequate vasoconstriction and a limited compensatory heart rate response during orthostasis, while marked recumbent hypertension may coexist.^[1-4]^ This phenotype creates a narrow therapeutic margin because interventions intended to improve upright BP may aggravate SH, whereas treatment directed at SH may worsen orthostatic symptoms or compromise upright perfusion.^[3,7]^

The principal diagnostic pitfall in this case was that the overall BP pattern was initially interpreted on the basis of persistently elevated seated BP before supine, standing, and 24-hour BP measurements had been characterized. Treatment intensification was followed by worsening orthostatic intolerance, recurrent syncope, injury, and marked loss of independence. Seated BP alone may fail to identify coexisting orthostatic hypotension, SH, and abnormal 24-hour BP patterns in patients with autonomic dysfunction.^[2,7]^ The deliberately cautious supine-to-seated-to-standing assessment documented profound orthostatic hypotension, presyncope, and only a modest increase in heart rate. In the context of a large orthostatic BP reduction, this limited chronotropic response was compatible with a neurogenic mechanism, although medication exposure and other clinical contributors also required consideration.^[5,6]^

Formal autonomic testing strengthened the etiologic interpretation by demonstrating combined cardiovagal and adrenergic dysfunction. Markedly reduced heart rate variability during deep breathing and the Valsalva maneuver was consistent with cardiovagal impairment, whereas inadequate and delayed BP recovery during the Valsalva maneuver was consistent with adrenergic dysfunction.^[2,6]^ In the absence of clinical features suggesting another primary neurodegenerative autonomic disorder, long-standing diabetes mellitus was considered the most likely underlying cause. Diabetic retinopathy supported the presence of established diabetic microvascular disease but was not considered diagnostic of autonomic neuropathy.^[2]^

Although autonomic testing supported a neurogenic mechanism, assessment for reversible or aggravating contributors remained essential before further treatment adjustment.^[3,9]^ No anemia, nutritional deficiency, electrolyte disturbance, thyroid dysfunction, renal dysfunction, overt heart failure, clinically relevant endocrine disorder contributing to BP instability, hemodynamically significant renal artery stenosis, or arrhythmia accounting for syncope was identified. Old lacunar ischemic lesions, hypertensive heart disease, mild aortic stenosis, and moderate obstructive sleep apnea remained clinically relevant because they influenced treatment selection and safety surveillance.

Nonpharmacological management remained the foundation of treatment. Recommended measures for nOH include optimization of fluid and salt intake, compression therapy, gradual postural transitions, physical counter-maneuvers, head-of-bed elevation, avoidance of prolonged standing, and education of both patients and caregivers.^[3,10,11]^ Smaller and more frequent meals may alleviate meal-related symptoms, although postprandial hypotension should ideally be confirmed by BP measurements obtained in relation to meals.^[6,10]^ In the present case, this nonpharmacological strategy was implemented comprehensively but was insufficient to restore adequate upright tolerance when used alone. The involvement of the patient’s wife was particularly important because severe symptoms, fear of falling, and loss of independence limited his ability to implement a complex treatment plan without assistance.

Supine and nocturnal hypertension required separate consideration because persistent nocturnal hypertension in autonomic failure may promote pressure natriuresis, nocturia, overnight volume loss, and worsening morning orthostatic hypotension.^[4,7]^ Sustained SH may be associated with long-term cardiac, renal, and cerebrovascular injury, although current evidence is insufficient to define treatment thresholds according to end-organ damage or mortality.^[4,7]^ The objective was therefore not complete normalization of nocturnal BP, but reduction of extreme hypertensive exposure without causing clinically important hypotension during the first morning mobilization.

Captopril 12.5 mg was initially introduced in the evening but aggravated orthostatic symptoms. Diltiazem 30 mg was subsequently introduced and was likewise not tolerated because of worsening orthostatic symptoms. A removable transdermal nitroglycerin patch was then selected to provide a time-limited antihypertensive effect while the patient was recumbent and was removed before morning mobilization. Short-acting nighttime antihypertensive strategies, including transdermal nitroglycerin, have been studied in autonomic failure, but the evidence base remains limited and treatment must be individualized.^[7]^ In this case, avoidance of nocturnal standing and use of a bedside urinal were individualized safety measures intended to reduce the risk of hypotension, syncope, and falls while the patch was active. Twenty-four-hour BP monitoring documented a substantial reduction in mean asleep BP after introduction of this time-limited nocturnal regimen, while daytime BP remained elevated and variable.

The selection of therapy for daytime orthostatic hypotension required particular caution. Midodrine is a short-acting α1-adrenergic agonist that increases vascular resistance and may improve standing BP and orthostatic symptoms, but it may also aggravate SH. It should therefore be timed to avoid late-day dosing and the supine position after administration.^[3,5]^ In the present patient, severe preexisting supine and nocturnal hypertension and previous lacunar ischemic lesions raised concern that additional α1-mediated vasoconstriction could further increase recumbent BP in the presence of established cerebrovascular disease. Midodrine was therefore deferred; this reflected individualized risk assessment rather than a general contraindication.

Droxidopa is a pharmacological option for symptomatic nOH and may improve orthostatic symptoms and standing BP in selected patients.^[3,5]^ As with other pressor therapies, supine BP requires careful monitoring, and droxidopa should not be administered near bedtime because SH may occur.^[3]^ Droxidopa was not available for clinical use in our setting.

In the present case, treatment was not selected according to a fixed hierarchy of pharmacological agents. Rather, persistently low oral fluid intake and bedside volume assessment raised clinical concern for relative volume depletion as a potentially modifiable contributor to upright hypotension. Given the coexisting severe supine and nocturnal hypertension, fludrocortisone was therefore introduced cautiously as a low-dose morning therapeutic trial, with predefined monitoring of orthostatic symptoms, upright tolerance, weight change, signs of fluid retention, serum electrolytes, supine BP, and clinical evidence of heart failure. This case-specific decision was supported by the absence of overt heart failure and a normal baseline N-terminal pro–B-type natriuretic peptide concentration. It should not be interpreted as evidence that fludrocortisone is generally preferable to droxidopa or midodrine. Fludrocortisone promotes sodium retention and intravascular volume expansion, but evidence regarding its effects on BP, orthostatic symptoms, and adverse events remains very uncertain, particularly beyond short-term treatment.^[12]^ It may be associated with weight gain, edema, hypokalemia, worsening SH, and heart failure decompensation. Observational data have associated its use with a higher rate of all-cause hospitalization than midodrine, particularly among patients with preexisting heart failure.^[12,13]^

The subsequent modest increase in body weight and development of peripheral edema were clinically relevant safety findings. Intermittent low-dose torasemide was used after discharge, while fludrocortisone was continued because symptomatic and functional improvement had been achieved. This case illustrates the challenge of balancing support of upright perfusion against the risk of fluid retention when both upright hypoperfusion and severe SH are present. Volume status may vary with oral fluid and salt intake, nocturnal pressure natriuresis, concomitant medications, and fluid losses related to sweating, ambient temperature, or physical activity. This variability complicates the adjustment of therapies that influence intravascular volume and thereby affect both OH and SH.^[3,8,12,13]^ Continued fludrocortisone treatment to support upright perfusion, combined with intermittent torasemide to manage edema, was an individualized response to emerging fluid retention and should not be interpreted as a standard regimen.

Once upright tolerance had improved, morning lercanidipine was introduced cautiously to reduce the remaining hypertensive burden. This reflected a shift in treatment priority: BP-lowering therapy was reintroduced only after severe orthostatic symptoms had improved and was evaluated in relation to standing tolerance, daily BP measurements, and 24-hour BP monitoring. In patients with hypertension and orthostatic hypotension, antihypertensive therapy should not automatically be withdrawn solely because orthostatic hypotension is present; treatment should be individualized according to symptoms, standing BP, comorbidity, and the overall BP pattern.^[8]^ In this case, the subsequent narrowing of the day–night BP difference did not represent restoration of a normal circadian BP pattern, because both mean awake and asleep BP remained elevated. Rather, it reflected attenuation of the previous extreme nocturnal predominance.

Serial 24-hour BP monitoring complemented clinical reassessment and postural BP measurement by documenting nocturnal hypertension, reverse dipping, and changes in awake and asleep BP after treatment adjustment.^[4,7]^ The measurements did not independently determine treatment decisions. Symptoms, functional status, standing tolerance, adverse effects, and patient safety remained equally important when treatment was adjusted.

Clinical response was therefore assessed across several clinical and hemodynamic domains rather than according to a uniform numerical BP target. Relevant outcomes included reduced orthostatic symptoms, absence of recurrent syncope and falls, improved upright tolerance, greater independence in daily activities, attenuation of extreme nocturnal hypertension, and identification of treatment-related adverse effects. Symptom relief and preservation of function are central therapeutic goals in neurogenic orthostatic hypotension; when severe SH coexists, therapies intended to improve upright BP should be adjusted cautiously, with attention to recumbent BP and the timing of administration.^[3,10]^

Moderate obstructive sleep apnea was a relevant comorbidity in this patient. Continuous positive airway pressure therapy was initiated after the initial symptomatic and BP improvement; therefore, obstructive sleep apnea treatment is unlikely to explain the early therapeutic response.

Several limitations should be acknowledged. As a single case report, this report cannot establish comparative efficacy or causality for individual components of the multimodal treatment strategy. Because interventions were introduced and adjusted over time as part of a multimodal regimen, the effects of nonpharmacological measures, fludrocortisone, transdermal nitroglycerin, and lercanidipine cannot be separated. No validated symptom scale, quality-of-life instrument, or standardized functional score was available. Clinical improvement was assessed on the basis of symptoms, absence of recurrent syncope or falls, upright tolerance, and independence in daily activities. The last documented outpatient assessment occurred approximately 4.5 months after hospital discharge, which limits assessment of longer-term recurrence of syncope or falls and longer-term clinical outcomes. Postprandial hypotension was not objectively documented.

A major strength of this case is the detailed longitudinal characterization of a clinically demanding hemodynamic phenotype through structured postural BP assessment, formal autonomic testing, serial 24-hour BP monitoring, functional status, and safety surveillance. The principal contribution is not the use of a particular drug, but the structured management of 2 competing physiological problems: inadequate upright perfusion and excessive recumbent BP. In patients with coexisting severe nOH and supine or nocturnal hypertension, a uniform BP target is insufficient. Treatment needs to be adapted to posture, time of day, symptoms, functional status, comorbidities, and treatment-related risks, with repeated reassessment of both clinical benefit and safety.

## 4. Conclusion

Severe nOH with coexisting supine and nocturnal hypertension in diabetic autonomic neuropathy requires assessment beyond a single BP value and management beyond a uniform treatment target. In this patient, integration of symptoms, functional status, postural BP responses, 24-hour BP patterns, comorbidities, and safety monitoring guided repeated, time- and posture-specific treatment adjustment and was associated with improved upright tolerance, increased functional independence, and attenuation of extreme nocturnal hypertension. Management requires repeated balancing of 2 competing priorities: protection from upright hypoperfusion and limitation of recumbent hypertensive burden.

## Author contributions

**Conceptualization:** Amela Kabaklić.

**Data curation:** Amela Kabaklić.

**Investigation:** Amela Kabaklić, Petra Šinigoj Porčnik.

**Methodology:** Amela Kabaklić, Petra Šinigoj Porčnik.

**Project administration:** Amela Kabaklić.

**Visualization:** Amela Kabaklić.

**Writing – original draft:** Amela Kabaklić, Petra Šinigoj Porčnik.

**Writing – review & editing:** Amela Kabaklić, Petra Šinigoj Porčnik.
